# Needle Trap Device-GC-MS for Characterization of Lung Diseases Based on Breath VOC Profiles

**DOI:** 10.3390/molecules26061789

**Published:** 2021-03-22

**Authors:** Fernanda Monedeiro, Maciej Monedeiro-Milanowski, Ileana-Andreea Ratiu, Beata Brożek, Tomasz Ligor, Bogusław Buszewski

**Affiliations:** 1 Interdisciplinary Centre of Modern Technologies, Nicolaus Copernicus University in Toruń, 4 Wileńska St., 87-100 Toruń, Poland; fmonedeiro@gmail.com (F.M.); milanowski.maciej@gmail.com (M.M.-M.); andreea_ratiu84@yahoo.com (I.-A.R.); bbusz@chem.umk.pl (B.B.); 2 “Raluca Ripan” Institute for Research in Chemistry, Babeş-Bolyai University, 30 Fântânele St., RO-400294 Cluj-Napoca, Romania; 3 Department of Environmental Chemistry and Bioanalytics, Faculty of Chemistry, Nicolaus Copernicus University in Toruń, 7 Gagarina St., 87-100 Toruń, Poland; 4 Department of Lung Diseases, Provincial Polyclinic Hospital in Toruń, 4 Krasińskiego St., 87-100 Toruń, Poland; bebro@wp.pl

**Keywords:** VOCs, NTD-GC-MS, breath, lung cancer, COPD, asthma, biomarkers

## Abstract

Volatile organic compounds (VOCs) have been assessed in breath samples as possible indicators of diseases. The present study aimed to quantify 29 VOCs (previously reported as potential biomarkers of lung diseases) in breath samples collected from controls and individuals with lung cancer, chronic obstructive pulmonary disease and asthma. Besides that, global VOC profiles were investigated. A needle trap device (NTD) was used as pre-concentration technique, associated to gas chromatography-mass spectrometry (GC-MS) analysis. Univariate and multivariate approaches were applied to assess VOC distributions according to the studied diseases. Limits of quantitation ranged from 0.003 to 6.21 ppbv and calculated relative standard deviations did not exceed 10%. At least 15 of the quantified targets presented themselves as discriminating features. A random forest (RF) method was performed in order to classify enrolled conditions according to VOCs’ latent patterns, considering VOCs responses in global profiles. The developed model was based on 12 discriminating features and provided overall balanced accuracy of 85.7%. Ultimately, multinomial logistic regression (MLR) analysis was conducted using the concentration of the nine most discriminative targets (2-propanol, 3-methylpentane, (*E*)-ocimene, limonene, *m*-cymene, benzonitrile, undecane, terpineol, phenol) as input and provided an average overall accuracy of 95.5% for multiclass prediction.

## 1. Introduction

Respiratory diseases are conditions which affect the airways and other structures of the lungs and they are represented by lung cancer, asthma, tuberculosis, chronic obstructive pulmonary disease (COPD) and pneumonia, being the leading causes of mortality and morbidity globally. Smoking or exposure to secondhand smoke is the main risk factor associated to most of respiratory diseases, with current smokers 11 times more likely to develop lung cancer compared to non-smokers [1]. Globally, respiratory diseases affect 1 billion people and account for 7% of all deaths worldwide. Nevertheless, even considering that lung cancer is one of the leading causes of death worldwide, COPD and asthma are predominant lung diseases that represent a burden on society in terms of health care costs [2]. The diagnosis of asthma or COPD is usually made by non-invasive techniques based on spirometry, however lung cancer is often diagnosed in late stages, due to the lack of noticeable clinical manifestations, or because these can be easily associated with other symptoms. This fact may reduce the chance of applying a timely and effective treatment. Currently used diagnostic methods for respiratory diseases includes physical examination followed by a set of chemical, imaging, endoscopic and immunological procedures [3]. Because different lung diseases are characterized by inflammation and other correspondent symptoms, direct assessment of airways may be applied, by using invasive procedures such as: computer tomography, bronchoscopy, bronchoalveolar lavage or biopsy. These are costly, time consuming and/or invasive procedures [2]. Consequently, a simple, reliable, low-cost and non-invasive test, able to achieve the diagnosis in real time (minutes up to hours), using a mere sample of exhaled breath in highly desired.

Therefore, fast detection and characterization of volatile organic compounds (VOCs) emitted from different biological matrices (breath, sweat, saliva, plasma, tissues, exudates, urine, etc.) as a tool for diagnosis was approached [4,5,6,7,8,9,10,11]. Breath tests are minimally invasive procedures, which are more easily accepted by the patients. An exhaled breath sample consists of VOCs and the breath aerosol [12]. Breath consists of almost 3000 compounds which are present in different combinations and quantities. Consequently, not only specific biomarkers, but the global VOC profile can be potentially associated to a characteristic fingerprint for each disease [2]. Exhaled breath is largely composed of nitrogen, oxygen, carbon dioxide, water, and inert gases. Trace components—volatile substances that are generated in the body or absorbed from the environment—present in the nmol/L–pmol/L (ppb volume—ppt volume) range make up the rest of the breath. The endogenous VOCs are generated by the cellular biochemical processes of the body, hence VOCs existent in human breath can reflect endogenous metabolic processes which occur in the tissues. VOCs-patterns in exhaled breath have been associated with various respiratory diseases such as cancer, asthma, COPD, cystic fibrosis, tuberculosis, etc. [13,14]. Breath samples are probably the most adequate to reach the rapid diagnosis of respiratory conditions, once substances from surrounding blood vessels and tissue can be exchanged in the alveoli and be available in the exhaled air. A large number of VOCs has been reported in scientific literature as markers of various diseases, as well as bacterial infections. These compounds can be divided into different chemical groups [15,16,17]: saturated hydrocarbons (stable end products of lipid peroxidation) and unsaturated hydrocarbons (e.g., from mevalonic pathway of cholesterol synthesis) [6,16], alcohols (which can be addressed as oxidized products of hydrocarbons and their precursors) [16], aldehydes (associated with inflammatory processes, resulting from lipid peroxidation) [5,18], ketones (products of fatty acid decarboxylation processes in the liver, associated to a diet rich in proteins and fat) [16], aromatic VOC–typically related to exogenous sources such as tobacco smoke and pollution [19], sulfur-containing compounds generated by incomplete metabolism of methionine in the transamination pathway and also associated with bacterial activity [20,21]), and nitrogen-containing compounds (such as ammonia, dimethylamine and trimethylamine, derived e.g., when conversion to urea is limited due to an impairment of liver function) [17].

Nowadays, gas chromatography–mass spectrometry (GC-MS) is considered a gold standard for VOC analysis [22]. Solid phase microextraction (SPME) or sorption tubes followed by thermal desorption are the most frequently used pre-concentration techniques in breath analyses. A prominent sampling tool is the needle trap device (NTD), which consists of a sorbent material packed inside a needle, working as an extraction trap [23]. This solventless technique provides exhaustive extraction and has potential for laboratory automation [24,25]. In the present work, NTD was used as extraction technique, followed by GC-MS analysis. VOCs were analyzed in breath samples belonging to healthy controls and patients with lung cancer, asthma and COPD, in an attempt to develop a classification model able to discriminate between these lung diseases, which have in common inflammatory processes in the lungs. In this sense, besides the assessment of global VOC profiles, 29 target compounds previously reported as potential biomarkers of the referred respiratory diseases were also investigated and quantified in breath samples.

The present study describes the non-invasive assessment of asthma, COPD and lung cancer, based on breath analysis of VOCs. Once all of these are lung diseases involving inflammatory mechanisms, the applied design of data analysis intended to find specific VOC patterns able to provide discrimination between these illnesses. The comparison between self-annotated discriminating features and compounds reported by literature as indicators of lung diseases represents an original approach for the validation of candidate biomarkers. The outline of the work presents the application of NTD for the determination of VOCs in breath. The found results aim to support the implementation of breath analysis to the clinical practice, as an accurate and reliable diagnostic tool.

## 2. Results and Discussion

### 2.1. Calibration Method and Quantitation of Analytes

Table S1 presents information regarding calibration method, while Table 1 displays data concerning the quantitation of analytes in breath samples. Obtained limits of quantitation (LOQs) ranged from 0.003 (3-methylpentane, 2-butanone, toluene, isododecane, 1,2,4-trimethylbenzene, (*E*)-ocimene, limonene, *m*-cymene and benzonitrile) to 6.21 ppbv (tridecane). Higher limits were obtained for heavier and more polar analytes, which also displayed wider linearity ranges. Lower limits were associated to compounds with higher volatility, a factor that seemed to contribute for their more efficient recovery, besides their expected greater stability in samples. Relative standard deviation (RSD%) did not exceed 10%, demonstrating that the proposed method provided adequate reproducibility. In general, suitability of NTD for preconcentration of analytes in gas mixtures could be inferred. Among the targets, isoprene and 1-propanol were found in each breath sample. Styrene, decane and phenol were observed in lowest frequency of appearance. Ethanol, isoprene and acetoin were the targets which occurred in higher concentrations in all sample’s cohorts. Carry-over effect was not observed, indicating that there is no influence of previously analyzed samples on the current ones.

### 2.2. VOCs Detected in Breath

**Figure 1:**
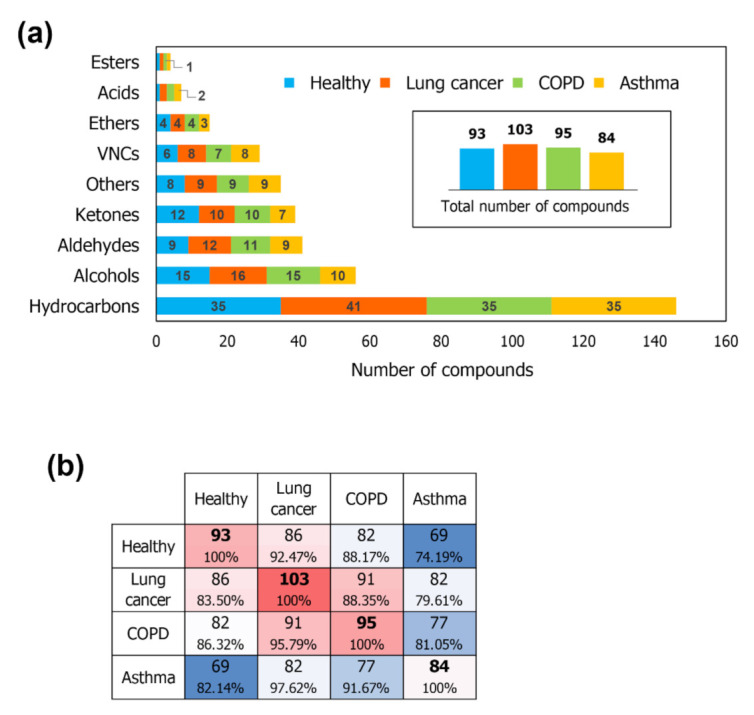
(**a**) VOCs distribution according to main chemical classes, in profiles belonging to the different studied groups, the contoured box displays the total number of compounds found in each group; (**b**) Similarity matrix displaying number and percentage of overlapping VOCs in the acquired profiles.

Regarding the obtained VOC global profiles, a total number of 112 different VOCs were detected. The VOCs most frequently observed in the samples were hydrocarbons, alcohols, aldehydes and ketones. A graph displaying the distribution of VOCs according to the functional groups in profiles belonging to the different studied groups is presented in Figure 1a. In general, the number of compounds belonging to each of the chemical classes seems to be proportional when evaluating the different studied conditions, however, some particularities of the qualitative composition of each group of profiles can be evidenced. Lung cancer and COPD profiles appear to be associated to a greater variety of compounds (103 and 95 detected VOCs, respectively), while asthma profiles are composed by smaller number of compounds (84 detected VOCs). An increased number of hydrocarbons is observed in the VOC composition in breath of lung cancer patients. Moreover, samples from patients with lung cancer and COPD appear related to a greater variety of aldehydes (12 and 11, respectively, against 9 found in healthy). This observation can be due to the fact that hydrocarbons and aldehydes are frequently reported as the most characteristic products of oxidative stress induced by inflammatory process [26,27,28].

A matrix displaying number and percentage of overlapping VOCs in the acquired profiles is presented in Figure 1b. By the content of coincident compounds, the level of similarity regarding the qualitative composition of breath samples can be inferred. In this sense, lung cancer and COPD profiles, display the greater similarity between each other, followed by the VOC profile of lung cancer and healthy individuals, while asthma breath samples present to be the most distinct in terms of composition.

### 2.3. Differential Distribution of VOCs

**Figure 2:**
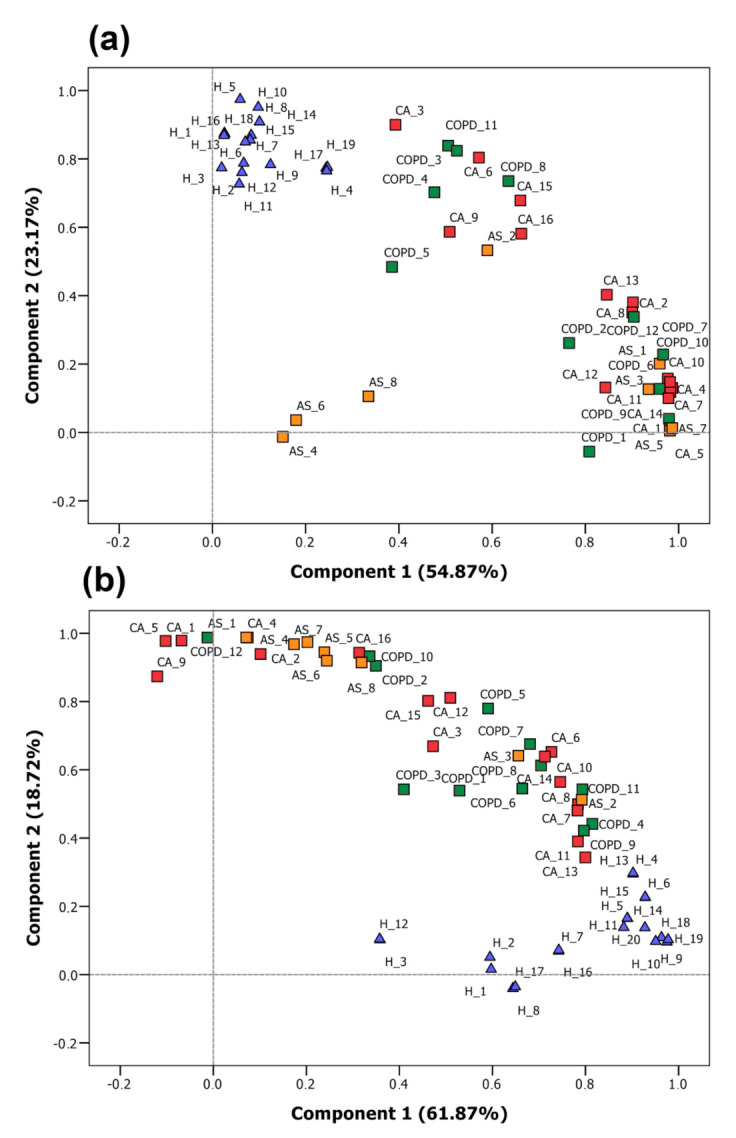
PCA plots using as input (**a**) VOCs’ responses in global profile analysis, (**b**) responses of the targets quantified in the samples (triangles = control samples; squares = positive samples).

Principal component analysis (PCA) was performed intending to identify relationships and existing patterns within datasets. Peak area data regarding the global VOC profiles was used as input for generation of the score plot depicted in Figure 2a, in which 78.04% of variance was represented by the two first principal components. When using as input the calculated concentration values of the 29 preselected analytes in samples, the plot presented in Figure 2b is produced. In this case, 79.72% of total variance was described by the components **1** and **2**. In both cases, around 80% of the total variance can be assigned to the observed distribution. Although both score plots indicate a discrimination between control cases and remaining samples, a clearer grouping can be observed when considering the global profile, once in Figure 1b control samples appear confined to an isolated cluster. Still, in both situations, the lack of a distinct grouping according to each of the investigated conditions demonstrates that other factors play a relevant role in the observed pattern of distribution of VOCs. This can be mainly related to the variability in the nature and extension of the involved pathophysiological mechanisms, inherent to the different lung diseases. Therefore, the usage of supervised approaches is essential to achieve the classification of samples in agreement with the related diagnosis.

**Figure 3:**
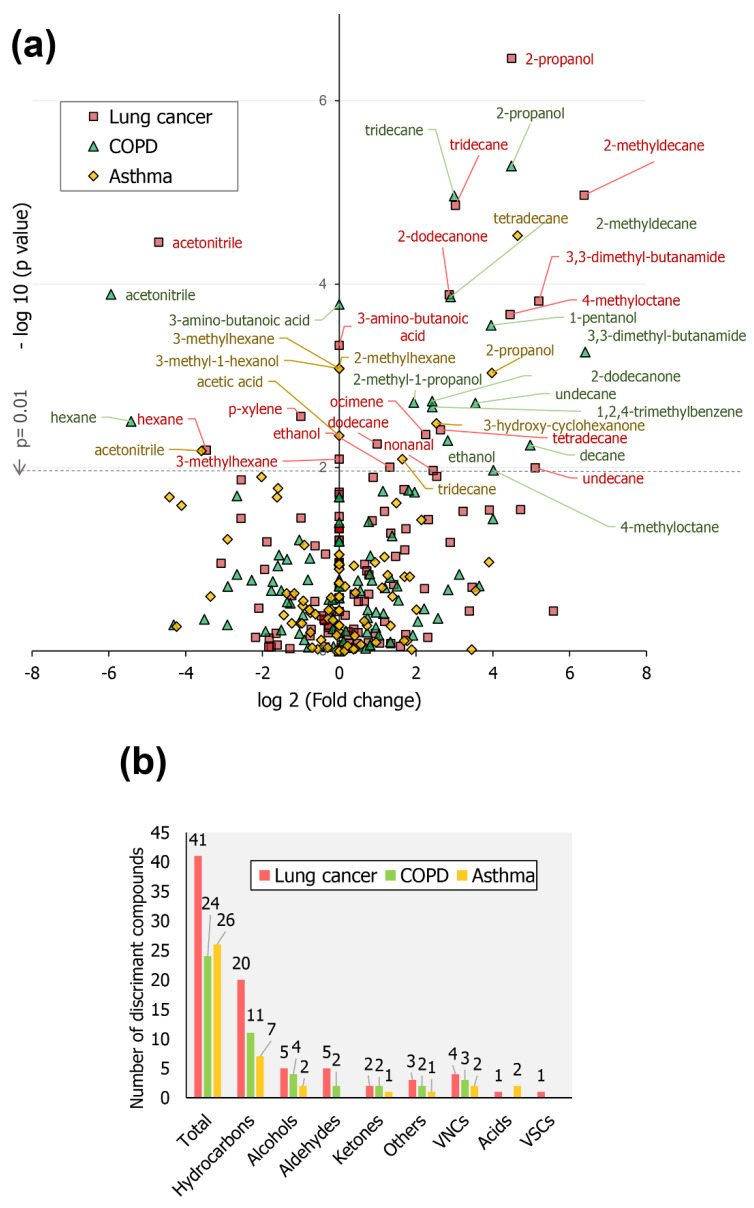
(**a**) Volcano plot displaying the most discriminating features, in terms of fold change (*x*-axis) and statistical relevance (*y*-axis), in which the dashed line represents the point of *y*-axis in which *p* = 0.01; (**b**) Graph of distribution of number of all compounds assigned as discriminating features, according to disease and chemical class (significance criteria: *p* ≤ 0.05).

A volcano plot was built in order to present found discriminating features when considering obtained global VOCs profiles. In Figure 3a the overall trend of the detected VOCs (variables) is graphically represented. The variables located above the dashed line refer to the compounds which displayed greater statistically relevant changes in their responses when compared to the control group. The variables located along the *y*-axis correspond to VOCs absent in the healthy group and detected solely in positive samples. In the left part of the plot are displayed compounds with decreased responses in the positive samples, while in the right side of the plot are displayed VOCs presenting an increased response in samples of diseased. The VOCs located towards the top of the graph expressed the greatest statistical significance. The names of the most discriminative components are exhibited in the plot.

Figure 3b presents a bar graph showing the distribution of all compounds classified as discriminant features, considering as criteria *p* ≤ 0.05. Most of the compounds which displayed significant alteration in their responses when compared to those presented in the healthy group belong to the class of hydrocarbons, followed by alcohols and aldehydes. In lung cancer profiles, a greater number of discriminating VOCs was verified (41 compounds). For asthma and COPD samples, 26 and 24 altered VOCs were indicated, respectively. As presented in Figure 1b, around 92, 88 and 74% of the compounds observed in lung cancer, COPD and asthma samples, respectively, were shown to be conserved in the healthy group profiles. This indicates that the differential abundance of VOCs in samples is determinant to discriminate between samples’ group, once the similarity between the qualitative profiles belonging to the four studied groups is not so divergent. Such observation highlights the importance of validated quantitative assays’ application regarding breath samples for diagnosis purposes.

Few compounds presented a more expressive incidence within the group of active smokers’ individuals, thus possibly being ascribed as products of cigarette smoke. 1,3-Cyclopentadiene was identified solely in this group, in 40% of the samples; 2,5-Dimethylfuran was detected in 80% of samples from active smokers, which represented around 73% of its total incidence across samples. Other substances commonly related to tobacco smoke composition, such as benzene and toluene [29], did not present a specific distribution within samples of smokers, probably because these can be originated from other various sources.

With respect to the VOCs found altered, acetonitrile is typically present in cigarette smoke, although also present in automobile exhaust and other anthropogenic emissions [30]. Considering that most of the enrolled subjects were not smokers, differentiated levels of this substance would not be expected. However, together with the decreasing trend observed for *p*-xylene, the reduction in the abundances of such compounds in positive group can be an indicative of diminished ability of elimination of exogenous through exhaled air, or a consequence of the augmented activity of cytochrome P450 isoforms documented in lung cancer [31], which could be responsible for the rapid metabolization of inhaled compounds in the lungs.

The two main lung cancer types are small-cell lung carcinoma (SCLC) and non-small-cell lung carcinoma (NSCLC). Two hypothesis involve SCLC histogenesis: the first assumes that SCLC derives from cells of the diffuse endocrine system, i.e., the amine precursor uptake decarboxylation (APUD)-system, the second suggests this type of lung cancer originates from the endodermbronchial lining [32,33]. Adenocarcinoma (NSCLC subtype) arises from glandular cells of bronchial mucosa, whereas squamous lung cancer origins from the modified bronchial epithelial cells and adenosquamous carcinoma contains two types of cells: squamous cells (thin, flat cells that line certain organs) and gland-like cells. Finally, large cell (undifferentiated) carcinoma originates from epithelial cells of the lung [32]. The origin and nature of the malignant cells is crucial for different treatment strategies. Tumor tissue releases different protein biomarkers according to subtype of cancer. The same concerns different types or amounts of certain VOCs secreted by various malignant part of cell. The oxidation of fatty acids present in the cell membranes is pointed out as the source of VOCs associated to oxidative stress condition. The mentioned process is initiated by the reactive oxygen species (ROS) which are found in increased levels in inflamed tissues [17,28]. 

Due to the ROS activity, mechanism chain reactions occur, with radicals tending to be stabilized through alpha and beta scissions [34], leading to the formation of a variety of shorter chain fatty acids, alkanes, alkenes, alcohols and aldehydes. In addition, formed compounds can be subjected to other reactions, aiming their transformation into smaller and more polar molecules [35]. Cancer cells are characterized by their enhanced metabolism and altered functions in several biochemical pathways [36]. Therefore, metabolite profile consisting of a greater variety of compounds may be expected. Hexane can be possibly formed during the oxidation of oleic acid [34], while can be addressed as an exogenous substance as well. Hexane showed decreased abundance in cancer and COPD samples. This fact can be explained by three hypotheses: impaired excretion through exhalation [37], enhanced conversion of the specie into oxidized forms [38] and favoring of alternative mechanism, which gives rise to different products, during lipid oxidation associated to oxidative stress particular to the referred conditions.

1-Pentanol can be interpreted as a pentane oxidation product, caused by cytochrome P450, and recognized as a metabolite of reactive oxygen species reactions with omega-6 fatty acids [26]. Methyl ketones such as 2-dodecanone can be formed by the decarboxylation of β-keto acids during the metabolism of fatty acids [39]. Nonanal can be also formed by different mechanisms during ROS attack on oleic acid from cell membranes [34]. Medium-chain branched alkanes, such as 2-methyldecane and 4-methyloctane, were pointed out by previous works as oxidative stress indicators [40,41]. However, their generation by human organism due to the oxidation of lipids is questionable, as cell membranes contain only linear chain lipids [26].

Branched alkanes can be originated from microbial lipids, mostly produced in the fatty acid pathway of bacteria, by using amino acids as precursor molecules which are submitted to elongation in this biochemical path [42,43]. Considering this, the occurrence of methylated branched alkanes in breath could be connected with bacterial activity. Alternatively, these could be products of transformation/degradation of prenyl molecules in organism, a mechanism that also remains undescribed. Aromatic species, such as *p*-xylene (decreased in COPD) and 1,2,4-trimethylbenzene (increased in COPD), are frequently addressed as pollutants, although also possibly formed by bacterial shikimate and related pathways [44].

Regarding the 29 compounds belonging the set of selected targets, 15 of them presented themselves as discriminating features (*p* < 0.05) when assessing solely controls against positive samples, all of them displaying increased concentration in the positive group. They were 2-propanol, 2-methylpentane, 3-methylpentane, 1-propanol, 2-butanone, styrene, isododecane, 1,2,4-trimethylbenzene, (*E*)-ocimene, *m*-cymene, phenol, undecane, dodecane, terpineol and tridecane. However, as demonstrated in the next section, compounds other than these displayed usefulness in the characterization of studied groups, presenting themselves as discriminating variables related to disease type. A combination of mechanisms involved in carcinogenesis, inflammatory processes and microbiota activity–which develop important role in pathogenesis of several diseases, may play a part in the alterations observed for certain compounds in breath samples.

The propionic acid formed during microbial fermentation and the propionyl-CoA generated during amino acids degradation enters in the propanoate metabolism, which takes place in the mitochondria and comprehend a series of reactions coupled with other pathways related to cell energetics. In microorganisms, 1-propanol is a product of propanoyl-CoA transformation [45], while 2-propanol can be formed by the reduction of acetone produced during the synthesis of ketone bodies [46]. 2-Butanone is a secondary ketone, therefore its origin can be associated to the β-oxidation of fatty acids. The acetyl-CoA units generated in this process fuel the citric acid cycle, supplying energy generation [47]. Terpenoids are very diverse natural products synthetized by plants, but also by bacteria. These metabolites are associated to the mevalonate and deoxyxylulose phosphate pathways [48,49]. Although their biosynthesis in human so far remain unknown, studies have reported terpenoid derivatives as potential cancer indicators. Considering this, increased concentration of compounds such as (*E*)-ocimene, *m*-cymene and terpineol can either be a consequence of deficient metabolic function impairing proper elimination of these substances coming from diet [50], an indicative of specific bacterial activity, or even a product of transformation of isoprenoids derivatives due to the dysregulated mevalonate pathway in human during carcinogenesis [51].

Isododecane is known as a synthetic chemical with several applications in the industry [52], without any identified biosynthetic pathway so far. Styrene is a constituent of polymers, nevertheless, there is evidence that some microorganisms can produce styrene using phenylalanine as precursor molecule [53]. On the other hand, phenol is often reported as product of bacterial catabolism of aromatic amino acid species previously documented as elevated in gastroesophageal neoplasms [54].

Their formation of the *n*-alkanes undecane, dodecane and tridecane can be related to the oxidation of lipids, more precisely, a formed alkoxyl radical undergoes scission, generating an alkyl radical which abstracts a hydrogen atom, turning into a stable alkane [17,26]. 2-Methylpentane and 3-methylpentane are other branched species possibly derive from the oxidation of branched chain fatty acids generated by bacteria.

### 2.4. Diagnosis Prediction–Global Profiles

Most of the studies comprising the detection of diseases based on VOC analysis in biological samples compare paired data from healthy and diseased groups. Many of the compounds addressed as candidate biomarkers by literature are explained as produced by oxidative stress–a process promoted by typical inflammatory immune responses and thus non-specific. In this sense, illnesses sharing common etiological and pathological processes may play a part as confounding factors when a specific diagnosis is intended. For this reason, the present and following sections of the manuscript were dedicated to the development of statistical models able to identify and discriminate specific VOC patterns, allowing simultaneous differentiation of the studied lung diseases.

**Figure 4:**
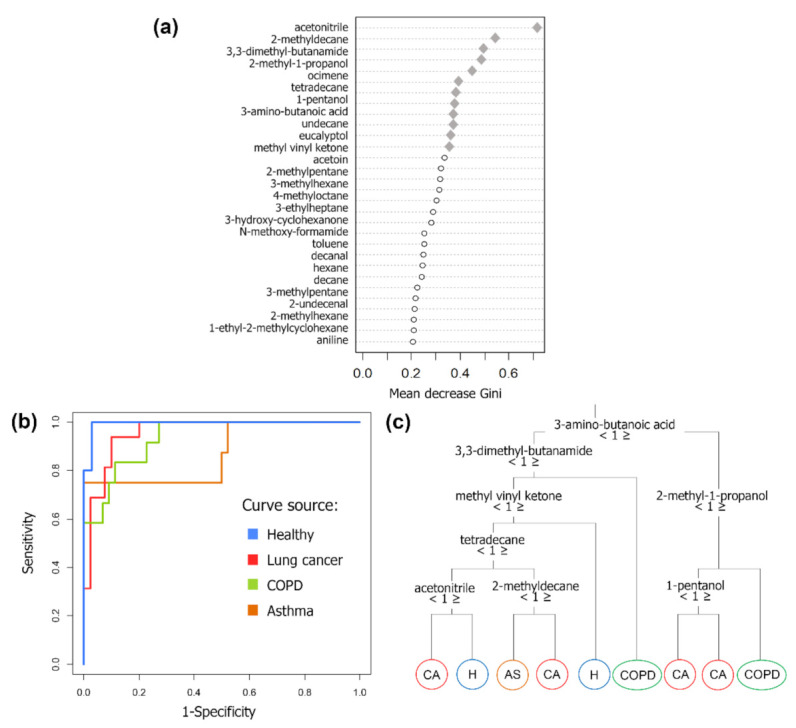
(**a**) Variable importance plot in terms of mean decrease Gini (node purity), obtained in the first training of RF model. Diamonds refer to VOCs selected for generation of the final classificatory model; (**b**) ROC curves based on RF’s final model output regarding the test set, using a panel of 12 VOCs; (**c**) Example decision tree produced by RF analysis, in which obtained accuracy was 81% (AS = asthma, CA = lung cancer, COPD = chronic obstructive pulmonary disease, H = healthy).

A random forest (RF) analysis was conducted on global profiles data, aiming to classify obtained VOC fingerprints into the four investigated categories. Variance importance was assessed based on the mean decrease Gini when one of the questioned variables is removed from a preliminary RF model. Gini impurity can be interpreted as the chance of a case sampled randomly to be incorrectly classified in relation to a given class, thus being related to the purity of cases within a tree node [55]. Therefore, greater decreases in this measurement indicate greater importance of a given variable. The resulting plot is presented in Figure 4a, the compounds are ranked from the most essential to those less relevant for the obtaining of homogenous classes. The 12 most important variables were assigned to compose the RF final model, the selected compounds appear depicted as the gray diamonds, in the upper part of the graph. The intention was to obtain the greater model overall accuracy as possible, including a minimum number of features.

Predict probabilities of a case of the validation set to belong to a class were provided by RF modeling. The receiver operating characteristic (ROC) curves presenting the ability of the model to predict a certain condition are showed in Figure 4b, information on parameters regarding classification performance are presented in Table 2. It can be observed that class recognition was performed with at least 93% of sensitivity and 87.5% of specificity for lung cancer, asthma and healthy groups. Regarding the later mentioned groups, prediction with accuracy above 87% was achieved. The lower prediction capability obtained in case of COPD (67%). An exemplary decision tree, from the 1000 generated during modeling, is presented in Figure 4c.

### 2.5. Diagnosis Prediction–Target Analysis

**Figure 5:**
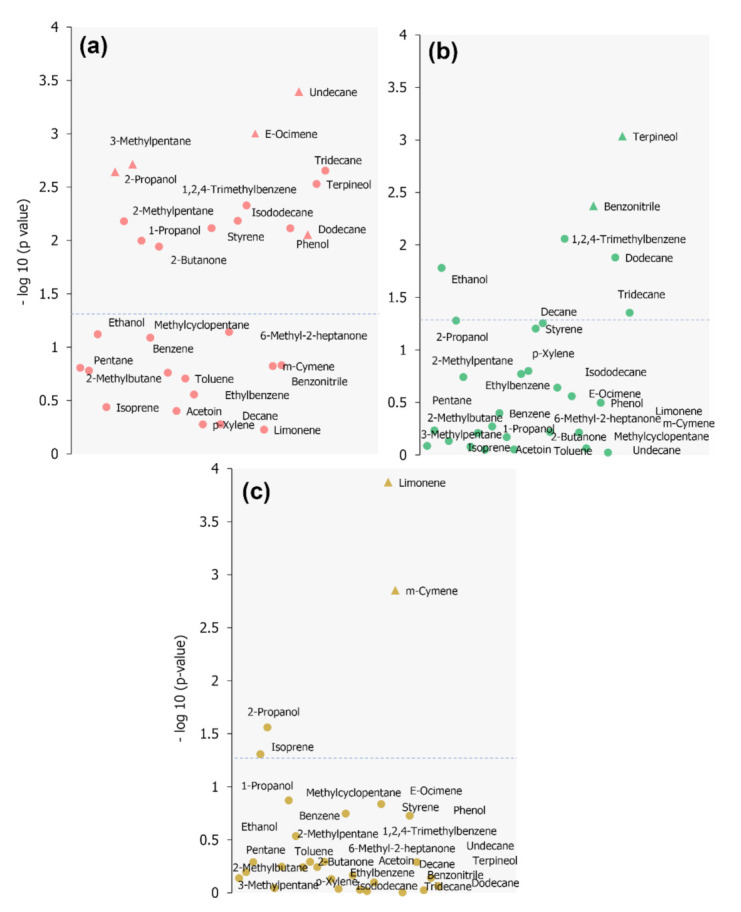
Plot of −(log _10_) of p values when applying Mann-Whitney test for specific classes: (**a**) lung cancer, (**b**) COPD or (**c**) asthma, against all other conditions. Dashed line represents where *p* ≤ 0.05. Variables represented by triangle shape icon were those included in MLR final model.

In this section, in accordance with the criteria described in the Material and Methods section and empiric observations drawn from multinomial logistic regression (MLR) performance using different set of variables, 2-propanol, 3-methylpentane, (*E*)-ocimene, limonene, *m*-cymene, benzonitrile, undecane, terpineol, phenol were the compounds selected to build the MLR final model. A clearer depiction of variables distribution according to their importance can be observed in Figure 5. Table 3 presents information regarding the developed model, which, when applied to the train and test datasets provided 100% and 90.5% of accuracy, respectively (average overall accuracy = 95.3%).

In MLR, coefficients can be multiplied by the quantitative inputs for the calculation of probabilities of a case to belong to a specific condition. Equation (1) presents the model regression equation, where ln[P/(1 − P)] represents the log-odds pertinent to a specific disease, β_0_ is the intercept and β_1…k_ are the coefficients provided by the MLR model, referring to the variables X (in the case, the concentration of the selected targets). A case for which the calculated probabilities are greater than 50%, can be assigned as belonging to that class.

ln[P/(1 − P)] = β _0_ + β _1_ · X _1_ + … + β _k_ · X _k _ (1)

**Figure 6:**
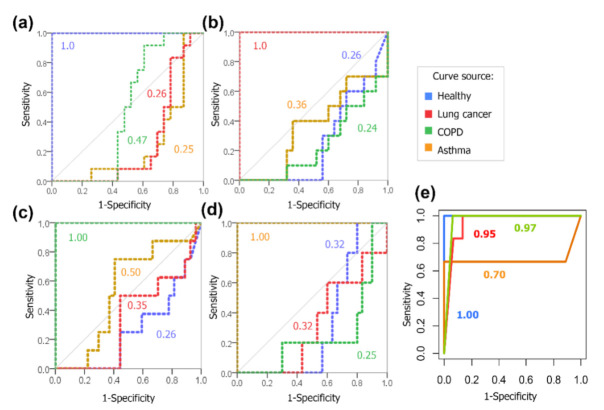
ROC curves generated from fitted values (train set) created by MLR model, labelling (**a**) healthy, (**b**) lung cancer, (**c**) COPD and (**d**) asthma as the state variables; (**e**) ROC curves generated from predictions computed by the MLR model for the test set. Colored numerals refer to values of AUC obtained for each depicted curve.

The numerical coefficients provided by MLR can be interpreted as weights, or the contribution of these variables to the designated classes. Positive coefficients are related to compounds with increased response when comparing to the reference class (“Healthy”), while negative coefficients are associated to targets which were present in lower concentrations in positive samples. In a closer interpretation, the coefficients express multinomial log-odds. For example, assuming that all other variables remain constant, an increase of one unit in the concentration of 2-propanol multiplies the odds of a sample belonging to the asthma group instead of healthy group by 0.56. On the other hand, an increase in one unit of (*E*)-ocimene concentration in breath implies the log-odds of COPD to decrease by 64.27, in an assumption that the remaining variables are kept constant. Considering this, increased levels of limonene and *m*-cymene are characteristic from samples of asthma patients, while increased level of undecane and decreased concentrations of benzonitrile are observed for breath of individual with lung cancer; Moreover, greater concentrations of phenol and lower concentrations of *m*-cymene are particularly observed in samples from COPD patients. Values fitted for the train set and predictions performed by MLR method solely for the validation set were used as input to build ROC curves (Figure 6a–d and Figure 6e, respectively). Values of area under the curve (AUC) presented in Figure 6a–d represent the probability of samples belonging to a given group to be classified as the state condition. For each class specified in the model, AUC was 1.0, meaning that 100% of sensitivity and specificity was obtained. On the other hand, cases not assigned as the state variable provided AUC ≤ 0.5 (curves below random guessing line). When considering the performance of the model on the test data, an overall accuracy of 91% was obtained, resulting in an average accuracy of 95.5% when both evaluated sets are considered. Detailed information regarding MLR performance is presented in Table 4.

## 3. Materials and Methods

### 3.1. Apparatus and Standards

The analyses were conducted on a model 6890 A gas chromatograph coupled with a 5975 Inert XL MSD (Agilent Technologies, Waldbronn, Germany). Inlet temperature was kept at 260 °C and carrier gas (helium 6.0) flow was set at 2.2 mL min^−1^. A DB-624 capillary column (Agilent) 60 m × 0.32 mm × 1.8 µm was used. The oven temperature program was as follows: initial temperature was 35 °C (held for 3 min), ramped to 50 °C, then 75 °C, 200 °C and finally 240 °C, at rates of 3 °C min^−1^, 5 °C min^−1^, 15 °C min^−1^ and 10 °C min^−1^, respectively. The last temperature was kept for 15 min, resulting in a run time of 41.33 min. Full scan spectra were acquired within a range of 30–300 *m*/*z*, at electron ionization (EI) of 70 eV. The ion source and transfer line were set to 250 °C. Chromatographic data acquisition was performed using MSD ChemStation E.02.00.493 software (Agilent). Compounds identification was performed using NIST05 mass spectra library. Each peak was searched manually, including baseline subtraction and averaging over a peak. Forward match quality of at least 750/1000 was applied as the lower match threshold.

Needle trap device 700-60d-PXC (PDMS + Carbopack X + Carboxen 1000) was purchased from PAS Technology (Magdala, Germany). The air pump flow was conducted by a sampling case model SC-B (PAS Technology), designed for controlled air sampling. Prior first use, NTDs were conditioned in a heated conditioner (PAS Technology) at 300 °C under helium flow (1 bar), for 30 min, in order to remove VOC’s contaminations from sorbent. One liter-Tedlar bags were obtained from SKC (Eighty Four, PA, USA).

Chemicals used as standards (2-methylbutane, pentane, ethanol, isoprene, 2-propanol, 2-methylpentane, 3-methylpentane, 1-propanol, methylcyclopentane, 2-butanone, benzene, acetoin, toluene, ethylbenzene, *p*-xylene, styrene, decane, 6-methyl-2-heptanone, isododecane, 1,2,4-trimethylbenzene, ocimene, D-limonene, *m*-cymene, benzonitrile, phenol, undecane, dodecane, terpineol and tridecane) were purchased from Sigma-Aldrich (St. Louis, MO, USA), all with purity > 98%.

### 3.2. Breath Collection

The study was approved by the local Ethics Committee of Collegium Medicum in Bydgoszcz (No. KB 621/2016–25.10.2016). Individuals aged over 18, with positive clinical diagnosis for lung cancer (non-small cell lung cancer, subtype: adenocarcinoma) (n = 16), chronic obstructive pulmonary disease (n = 12) and asthma (n = 8) were recruited at the Department of Lung Diseases of the Provincial Polyclinic Hospital in Toruń. Samples from enrolled cancer patients were obtained before any medical intervention (such as neoadjuvant therapies or surgery).

Individuals were refrained to eat, drink or smoke 2 h prior sample collection. No special dietary regimes were applied. All individuals gave informed consent to participation in the study. The patients completed a questionnaire describing their age, gender and current smoking status (active smokers, non-smokers). Samples of mixed alveolar breath gas (alveolar and dead space gas) were collected in Tedlar bags with parallel collection of ambient air at the same room. Breath samples were obtained after approximately after 10 min rest in the same ambient. Each subject provided breath samples using a disposable plastic straw connected to the Tedlar bag.

Control samples (n = 20) were collected from healthy individuals aged over 18 years, without any history of positive diagnosis for cancer or respiratory diseases, who were not suffering from any other inflammatory disease. All samples were analyzed within 2–3 h after collection–this timeframe was considered adequate to avoid the interference of gas composition losses [56]. In the total, 56 breath samples were collected. Information regarding enrolled volunteers is summarized in Table 5 (details regarding presented significance probabilities are described in the section “Data analysis and chemometrics approaches”).

Tedlar bags involved in sample collection and calibration experiments were daily treated with several cycles of cleaning, each consisting of consecutively filling and evacuating argon 5.0 from the bag. Afterwards, the bags filled with argon were kept in an oven at 65 °C. The content bag was tested before breath sampling, by means of GC-MS, in order to verify the effectiveness of cleaning procedure.

### 3.3. Selection of Targets

The compounds selected as targets were VOCs previously reported as potential breath biomarkers of lung cancer, COPD and asthma, in accordance with previous studies on this theme. A literature search was performed in the electronic database Web of Science Core Collection (from Clarivate Analytics; Philadelphia, PA, USA), as well as Google Scholar. The searched terms were: “volatile organic compounds”, “gas chromatography”, “biomarker”, “lung cancer”, “COPD” and “asthma”, considering a time span from 1999 to 2016. The indexed literature is presented in the Supplementary Material (Table S2) [57,58,59,60,61,62,63,64,65,66,67,68,69,70,71,72,73,74,75,76,77,78,79,80].

### 3.4. Calibration Procedure

Gas mixtures of the analytes were prepared by injection of 1 µL of liquid standards into 1 L glass bulb (Supelco, Bellefonte, PA, USA) previously evacuated. Methanol HPLC was used for the preparation of 50:50 (*v*/*v*) dilution of acetoin, phenol and terpineol, which are solids at room temperature. After the complete vaporization of the liquids, the interior of the bulb was equilibrated with nitrogen, generating a gas mixture of the compounds of interest. Using a gas-tight syringe, different volumes of the stock gas solution were transferred to Tedlar bags filled with 1 L of nitrogen, in order to obtain the desired concentrations.

The concentrations were calculated in terms of part per billion per volume of analyte (ppbv), taking in consideration their molar volume. Six calibration levels were used in the construction of calibration curves, all analyzed in triplicates. The limit of detection (LOD) was defined as the lowest detectable concentration of analyte, considering a signal-to-noise (S/N) ratio of at least 3. LOQ was considered as the lowest concentration of analyte with imprecision of at least 15%, considering a minimum S/N value equal to 10. Calibration was conducted by linear regression analysis, using the obtained experimental data. Linearity was evaluated by the method of least squares and reported as the coefficient of determination (R^2^). Linearity was confirmed for values of R² above 0.99. Inter-assay imprecision was assessed from the evaluation of assays in triplicate, these were expressed in terms of relative standard deviation (RSD%). Reported RSD% values are the average of imprecision calculated for lower (LOQs), medium (5.17–17.25 ppbv) and high calibration levels (9.52–3452.0 ppbv)—which concentrations varied depending on the linearity range displayed by the analyte.

### 3.5. Sample Extraction

Prior to sample extraction process, NTDs were conditioned for 10 min, at 300 °C, under helium 6.0 flow (Air Products, Warsaw, Poland). Samples in Tedlar bags were drawn through the air pump, at a flow rate of 30 mL min^−1^. The fixed volume of 50 mL was sampled from each bag. Once extraction was complete, the loaded NTD was desorbed into GC inlet port for 2 min.

### 3.6. Data Analysis and Chemometrics Approaches

For the building of main dataset, area of peaks belonging to ambient air samples were subtracted from respective samples obtained from patients. Evaluation of normality of distributions was conducted using Kolmogorov-Smirnov test. Differences between volunteers’ ages was assessed by t-test. Principal component analysis was performed in order to evaluate data patterns regarding sample’s group. Mann-Whitney test was used to indicate VOCs which presented statistically relevant differences in their responses in the studied groups, *p* ≤ 0.05 was considered as the relevance criteria. For the above cited tests, IBM SPSS Statistics v. 24 software (IBM Corp., Armonk, NY, USA) was used. The following approaches were executed in R environment, using RStudio console v. 1.1.463 (RStudio, PBC, Boston, MA, USA). Significant differences between the proportions of volunteers assigned to a certain group were assessed by the test of equal or given proportions, employing the R function “prop.test”. For chemometrics approaches, the packages “gplots”, “RandomForest”, “caret”, “ROCR” were employed. Random forest is a machine learning method based on recognition of latent patterns within global VOC profiles. RF was conducted in order to develop a multiclass model, able to distinguish between studied conditions. RF input consisted of peak table data converted into binary entries–once this algorithm was dedicated to non-quantified data, this format of dataset was considered as more appropriate than to express RF outcome in terms of peak area. Variable importance plots were assessed for selection of variables to be included in the model. Half of the data was randomly selected to compose the training set (bootstrap sampling method) and the remaining data was applied in the validation process. Receiver operating characteristic curves were built based on calculated probabilities output from RF modeling. Ultimately, the development of a classificatory model based solely on target compounds was aimed, for that, variables (targets) were selected according to their discriminative potential between all four investigated conditions. The criteria comprised most unique targets which presented higher discriminative relevance when considering a given condition against all others. MLR was performed using the package “nnet”, employing the data regarding quantitation of the selected targets in analyzed samples. This multiclass categorical method performs a linear combination of features, allowing prediction through the calculated probabilities of an input (set of features’ values) to belong to each class specified in the model. Sixty percent of the data regarding quantitation of targets in the samples was randomly addressed as the training set, while the remaining data was addressed to a testing set. “Healthy” group was defined as the reference class. ROC curves were prepared based on the predictions computed by developed MLR model for fitted values and test data.

## 4. Conclusions

The developed NTD-GC-MS method was demonstrated to be suitable for the determination of target VOCs in breath samples, providing considerably low limits of detection and quantitation, as well as appropriate reproducibility. From the 29 targets selected from literature, more than half of them presented significant differentiated responses among control and positive groups – found discriminating features were 2-propanol, 2-methylpentane, 3-methylpentane, 1-propanol, 2-butanone, styrene, isododecane, 1,2,4-trimethylbenzene, (*E*)-ocimene, *m*-cymene, phenol, undecane, dodecane, terpineol and tridecane, limonene and benzonitrile (which proved to serve for further differentiation between diseases). Built statistical models (using both self-annotated discriminating variables and quantified targets) aimed to simultaneously classify VOC profiles into lung cancer, COPD or asthma cases. Both classification models (RF and MLR), provided an overall accuracy above 80%. The distinction between VOC profiles related to clinical conditions involving concomitant molecular mechanisms is extremely relevant in order to assess cofounding aspects of breath analysis diagnosis. In this sense, machine learning tools and other mathematical models can be useful to identify disease-specific latent patterns. Cross-validated studies, comparing candidate biomarkers found by different research groups by means of different techniques, are essential for a future implementation of breath screening tests in a clinical setting. Such an approach can also enable a focused investigation of the pathways involved in the modulation of these potential biomarkers, as well as it can contribute to the establishment of optimized analysis protocols.

## Figures and Tables

**Table 1:** Data regarding quantitation of the targets in breath samples (H = healthy, CA = lung cancer, COPD = chronic obstructive pulmonary disease, AS = asthma, SD = standard deviation, nd = not detected, (−) = SD not calculated because analyte was quantified just in a single sample, nd = not detected).

Analyte	Average Concentration (ppbv)								Frequency in Samples (%)				
	H (SD)		CA (SD)		COPD (SD)		AS (SD)		Total	H	CA	COPD	AS
2-Methylbutane	1.52	(1.32)	3.73	(6.15)	1.63	(0.50)	1.72	(1.85)	37.5	25.0	50.0	33.3	50.0
Pentane	1.66	(0.67)	2.21	(1.09)	1.87	(0.53)	2.11	(2.26)	51.8	45.0	62.5	41.7	50.0
Ethanol	70.60	(95.14)	179.08	(132.87)	218.64	(216.1)	100.89	(108.96)	98.2	95.0	93.8	100.0	100.0
Isoprene	32.85	(34.01)	34.19	(30.08)	34.61	(20.9)	48.70	(16.95)	100.0	95.0	100.0	100.0	100.0
2-Propanol	10.55	(9.30)	230.66	(190.62)	258.37	(255.01)	123.42	(67.37)	85.7	55.0	100.0	100.0	100.0
2-Methylpentane	1.24	(0.30)	3.44	(2.41)	2.61	(2.07)	4.59	(5.57)	55.4	25.0	75.0	75.0	50.0
3-Methylpentane	0.24	(0.12)	0.93	(0.72)	1.27	(0.49)	1.07	(1.25)	35.7	10.0	68.8	33.3	25.0
1-Propanol	14.59	(14.63)	34.10	(37.73)	28.15	(38.54)	9.94	(5.77)	100.0	95.0	100.0	100.0	100.0
Methylcyclopentane	1.80	(0.53)	2.49	(1.11)	2.20	(0.47)	2.20	(0.27)	87.5	75.0	93.8	83.3	100.0
2-Butanone	1.74	(1.15)	1.93	(1.30)	1.45	(1.00)	1.26	(0.80)	80.4	55.0	100.0	83.3	87.5
Benzene	1.13	(0.83)	0.29	(−)	0.57	(−)	0.60	(0.09)	16.1	25.0	6.3	8.3	25.0
Acetoin	44.02	(19.8)	60.39	(51.63)	55.22	(28.95)	41.72	(17.93)	53.6	45.0	56.3	50.0	75.0
Toluene	6.23	(8.38)	0.98	(1.40)	0.63	(0.42)	0.89	(0.60)	55.4	40.0	75.0	58.3	50.0
Ethylbenzene	0.650	(0.65)	2.73	(2.50)	0.34	(0.36)	1.41	(−)	17.9	5.0	25.0	33.3	12.5
*p*-Xylene	1.15	(0.92)	1.62	(1.86)	1.40	(1.11)	1.97	(0.95)	41.1	25.0	50.0	58.3	37.5
Styrene	0.27	(0.26)	3.78	(6.26)	1.61	(1.30)	0.73	(0.59)	53.6	5.0	81.3	75.0	87.5
Decane	nd	(−)	nd	(−)	0.23	(−)	nd	(−)	1.8	0.0	0.0	8.3	0.0
6-Methyl-2-heptanone	1.65	(−)	4.42	(2.85)	1.72	(−)	6.46	(−)	12.5	5.0	25.0	8.3	12.5
Isododecane	0.69	(0.49)	1.59	(1.57)	0.98	(0.48)	0.52	(0.35)	76.8	45.0	93.8	83.3	100.0
1,2,4-Trimethylbenzene	0.83	(0.61)	2.55	(1.94)	2.60	(2.46)	1.42	(0.76)	82.1	50.0	93.8	100.0	100.0
(*E*)-Ocimene	1.16	(0.80)	4.64	(4.03)	2.95	(1.99)	2.98	(1.54)	82.1	50.0	100.0	91.7	100.0
Limonene	1.57	(1.20)	1.87	(1.80)	1.71	(1.75)	5.05	(2.15)	89.3	75.0	93.8	91.7	100.0
*m*-Cymene	0.61	(0.21)	0.41	(0.35)	0.38	(0.23)	0.32	(0.21)	46.4	10.0	62.5	50.0	87.5
Benzonitrile	1.44	(1.23)	3.57	(3.95)	4.57	(2.64)	2.12	(1.67)	78.6	50.0	93.8	91.7	87.5
Phenol	nd	(−)	52.78	(47.13)	75.02	(72.16)	nd	(−)	16.1	0.0	37.5	25.0	0.0
Undecane	0.80	(0.11)	3.83	(3.09)	2.44	(1.38)	1.78	(0.30)	41.1	20.0	75.0	41.7	25.0
Dodecane	5.18	(0.72)	10.58	(8.66)	9.51	(7.18)	6.27	(3.19)	73.2	45.0	87.5	91.7	87.5
Terpineol	3.57	(0.30)	17.36	(21.72)	26.53	(36.34)	6.87	(6.38)	71.4	15.0	100.0	100.0	100.0
Tridecane	3.43	(1.69)	42.16	(38.89)	28.36	(21.59)	8.59	(7.87)	51.8	10.0	75.0	75.0	75.0

**Table 2:** RF model performance (AUC = area under the curve, CI = confidence interval).

Statistics by Class	Sensitivity	Specificity	AUC	Balanced Accuracy
Asthma	75.0%	100%	0.872	87.5%
Lung cancer	93.8%	87.5%	0.956	90.6%
COPD	67.0%	97.7%	0.935	82.2%
Healthy	95.0%	94.5%	0.994	94.7%
RF overall accuracy (95% CI)	85.7% (73.7–93.6)			

**Table 3:** Description of MLR model (AS = asthma, CA = lung cancer, COPD = chronic obstructive pulmonary disease, SE = standard error).

Condition	Coefficients									
	Intercept	2-Propanol	3-Methyl-pentane	(*E*)-Ocimene	Limonene	*m*-Cymene	Benzonitrile	Undecane	Terpineol	Phenol
AS	−557.79	0.56	17.03	50.39	100.40	212.50	−22.69	−88.94	32.33	−12.95
(SE)	(42.62)	(0.37)	(2.55)	(2.48)	(10.34)	(11.52)	(1.32)	(3.64)	(1.31)	(5.31)
CA	−127.12	0.61	−129.32	32.92	32.33	31.11	−96.07	26.50	32.81	−1.89
(SE)	(29.25)	(0.38)	(17.43)	(8.11)	(9.90)	(6.47)	(16.91)	(11.17)	(8.40)	(0.86)
COPD	−23.49	0.49	−269.07	−64.27	11.09	−72.04	−7.72	−111.52	37.91	1.73
(SE)	(3.35)	(0.83)	(6.92)	(8.57)	(3.39)	(0.66)	(1.29)	(4.22)	(8.18)	(0.65)

**Table 4:** MLR model performance (AUC = area under the curve, CI = confidence interval).

Statistics by Class	Sensitivity	Specificity	AUC	Balanced Accuracy
Asthma	68.0%	100%	0.700	83.4%
Lung cancer	83.4%	93.4%	0.950	88.4%
COPD	100%	94.1%	0.971	97.1%
Healthy	100%	100%	1.000	100%
MLR overall accuracy (95% CI)	91.0% (70.0–99.0)			

**Table 5:** Main information regarding volunteers (SD = standard deviation, CA = lung cancer, COPD = chronic obstructive pulmonary disease, AS = asthma).

Group		Control		Positive		*p*
		n	%	n	%	
**Total**		20		36		0.367
**Gender**	Male	13	65.0%	27	75.0%	0.325
	Female	7	35.0%	9	25.0%	0.437
**Age (SD)**		41.2 (10.1)		66.8 (8.22)		0.078
**Smoking status**	Active smoker	2	10.0%	5 (2 COPD, 3 CA)	13.9%	0.287
	Ex-smoker	2	10.0%	22 (12 CA, 10 COPD)	61.1%	0.083
	Non-smoker	16	80.0%	9 (1 CA, 8 AS)	25.0%	0.640
**Condition**	Lung cancer	−		16	44.4%	
	COPD	−		12	33.3%	−
	Asthma	−		8	22.2%	

## Data Availability

Data is contained within the article or Supplementary Material.

## Supplementary Materials

The following are available online, Table S1: Data regarding calibration method of gas mixtures (LOD = limit of detection, LOQ = limit of quantitation, ppbv = part per billion per volume, R² = determination coefficient, RSD = relative standard deviation). Table S2: References which reported the targets selected in the present study as potential biomarker of lung diseases in breath samples, where: LC–lung cancer; COPD–chronic obstructive pulmonary disease.

## Sample Availability

Samples of the compounds are not available from the authors.
