# Supplementary material for: Needle Trap Device-GC-MS for Characterization of Lung Diseases Based on Breath VOC Profiles
Source: Molecules. 2021 Mar 22;26(6):1789. doi: 10.3390/molecules26061789 (PMC8004837; doi:10.3390/molecules26061789)
Supplement: Supplementary file 1 [file molecules-26-01789-s001.pdf]

NTD-GC-MS for characterization of lung diseases based on breath VOC profiles – Supplementary Material

Table S1 - Data regarding calibration method of gas mixtures (LOD = limit of detection, LOQ = limit of quantitation, ppbv = part per billion per volume, R^2 = determination coefficient, RSD = relative standard deviation).

**Analyte**	**LOD**	**LOQ**	**Linearity range**	**R^2**	**RSD (%)**
	**(ppbv)**				
2-Methylbutane	0.010	0.03	0.03-17.21	0.997	7.81
Pentane	0.105	0.315	0.315-17.49	0.990	7.60
Ethanol	0.430	1.29	1.29-3452.0	0.996	6.27
Isoprene	0.250	0.75	0.75-1886.0	0.993	10.00
2-Propanol	1.630	4.89	4.89-2616.0	0.992	9.61
2-Methylpentane	0.190	0.57	0.57-15.27	0.991	7.74
3-Methylpentane	0.001	0.003	0.003-15.43	0.996	6.95
1-Propanol	1.680	5.04	5.04-26.95	0.998	0.98
Methylcyclopentane	0.220	0.66	0.66-17.91	0.999	7.75
2-Butanone	0.001	0.003	0.003-22.5	0.996	7.29
Benzene	0.140	0.42	0.42-22.60	0.993	10.00
Acetoin	0.280	0.84	0.84-144.68	0.998	9.01
Toluene	0.001	0.003	0.003-19.03	0.991	6.17
Ethylbenzene	0.105	0.315	0.315-16.44	0.991	8.36
p-Xylene	0.100	0.30	0.30-16.32	0.991	7.45
Styrene	0.110	0.33	0.33-17.59	0.992	1.93
Decane	0.125	0.375	0.375-10.34	0.992	9.63
6-Methyl-2-heptanone	0.160	0.48	0.48-12.75	0.993	4.24
Isododecane	0.001	0.003	0.003-8.87	0.990	9.34
1,2,4-Trimethylbenzene	0.001	0.003	0.003-14.75	0.990	1.80
(E)-Ocimene	0.001	0.003	0.003-11.83	0.991	6.15
Limonene	0.001	0.003	0.003-12.44	0.990	6.45
m-Cymene	0.001	0.003	0.003-12.93	0.999	1.21
Benzonitrile	0.001	0.003	0.003-19.56	0.990	4.95
Phenol	1.430	4.29	4.29-11.46	0.993	9.48
Undecane	0.120	0.36	0.36-9.52	0.999	6.44
Dodecane	1.380	4.14	4.14-110.93	0.995	9.65
Terpineol	1.020	3.06	3.06-18.31	0.995	7.97
Tridecane	2.070	6.21	6.21-103.31	0.994	9.30

Table S2 - References which reported the targets selected in the present study as potential biomarker of lung diseases in breath samples, where: LC – lung cancer; COPD – chronic obstructive pulmonary disease [52,53,62–71,54,72–75,55–61].

Compound	Studied disease	Reference no. in Supplementary Material
2-Methylbutane	LC	[1]
Pentane	LC and COPD/COPD/LC	[2–4]
Ethanol	LC	[5]
Isoprene	LC/COPD/LC/LC and COPD/LC	[1,2,5,6]
2-Propanol	LC	[7]
2-Methylpentane	COPD/ Asthma/ LC and COPD/LC/LC	[2,4,8–10]
3-Methylpentane	LC	[4]
1-Propanol	LC	[1,4,11,12]
Methylcyclopentane	LC	[10]
2-Butanone	LC/COPD/LC	[1,4,8]
Benzene	COPD/Asthma/COPD/COPD/LC/ LC and COPD/LC	[2,8,10,11,13–15]
Acetoin	LC/LC	[1,16]
Toluene	COPD/COPD/LC and COPD/Asthma	[2,6,8,17]
Ethylbenzene	LC/LC and COPD/LC	[2,5,7]
p-Xylene	LC and COPD/LC/COPD	[2,13,18]
Styrene	LC and COPD/LC/LC	[2,10,19]
Decane	LC and COPD/LC/COPD	[2,10,20]
6-Methyl-2-heptanone	Wheeze	[21]
Isododecane	LC and COPD/LC	[2,10]
1,2,4-Trimethylbenzene	LC	[10]
E-Ocimene	COPD	[22]
Limonene	COPD	[20]
m-Cymene	Asthma	[23]
Benzonitrile	COPD	[22]
Phenol	COPD	[6,13]

Undecane	LC/COPD/LC	[1,19,22]
Dodecane	LC	[24]
Terpineol	COPD	[22]
Tridecane	COPD	[13]

References

1. Bajtarevic, A.; Ager, C.; Pienz, M.; Klieber, M.; Schwarz, K.; Ligor, M.; Ligor, T.; Filipiak, W.; Denz, H.; Fiegl, M.; et al. Noninvasive detection of lung cancer by analysis of exhaled breath. *BMC Cancer* **2009**, *9*, 348, doi:10.1186/1471-2407-9-348.

2. Poli, D.; Carbognani, P.; Corradi, M.; Goldoni, M.; Acampa, O.; Balbi, B.; Bianchi, L.; Rusca, M.; Mutti, A. Exhaled volatile organic compounds in patients with non-small cell lung cancer: cross sectional and nested short-term follow-up study. *Respir. Res.* **2005**, *6*, 71, doi:10.1186/1465-9921-6-71.

3. Fens, N.; Zwinderman, A.H.; van der Schee, M.P.; de Nijs, S.B.; Dijkers, E.; Roldaan, A.C.; Cheung, D.; Bel, E.H.; Sterk, P.J. Exhaled Breath Profiling Enables Discrimination of Chronic Obstructive Pulmonary Disease and Asthma. *Am. J. Respir. Crit. Care Med.* **2009**, *180*, 1076–1082, doi:10.1164/rccm.200906-0939OC.

4. Ligor, M.; Ligor, T.; Bajtarevic, A.; Ager, C.; Pienz, M.; Klieber, M.; Denz, H.; Fiegl, M.; Hilbe, W.; Weiss, W.; et al. Determination of volatile organic compounds in exhaled breath of patients with lung cancer using solid phase microextraction and gas chromatography mass spectrometry. *Clin. Chem. Lab. Med.* **2009**, *47*, 550–560, doi:10.1515/CCLM.2009.133.

5. Ulanowska, A.; Kowalkowski, T.; Trawińska, E.; Buszewski, B. The application of statistical methods using VOCs to identify patients with lung cancer. *J. Breath Res.* **2011**, *5*, 046008, doi:10.1088/1752-7155/5/4/046008.

6. Phillips, C.O.; Syed, Y.; Parthaláin, N. Mac; Zwiggelaar, R.; Claypole, T.C.; Lewis, K.E. Machine learning methods on exhaled volatile organic compounds for distinguishing COPD patients from healthy controls. *J. Breath Res.* **2012**, *6*, 036003,

doi:10.1088/1752-7155/6/3/036003.

7. Rudnicka, J.; Kowalkowski, T.; Ligor, T.; Buszewski, B. Determination of volatile organic compounds as biomarkers of lung cancer by SPME–GC–TOF/MS and chemometrics. *J. Chromatogr. B* **2011**, *879*, 3360–3366, doi:10.1016/j.jchromb.2011.09.001.

8. Fens, N.; Roldaan, A.C.; van der Schee, M.P.; Boksem, R.J.; Zwinderman, A.H.; Bel, E.H.; Sterk, P.J. External validation of exhaled breath profiling using an electronic nose in the discrimination of asthma with fixed airways obstruction and chronic obstructive pulmonary disease. *Clin. Exp. Allergy* **2011**, *41*, 1371–1378, doi:10.1111/j.1365-2222.2011.03800.x.

9. Smolinska, A.; Klaassen, E.M.M.; Dallinga, J.W.; van de Kant, K.D.G.; Jobsis, Q.; Moonen, E.J.C.; van Schayck, O.C.P.; Dompeling, E.; van Schooten, F.J. Profiling of Volatile Organic Compounds in Exhaled Breath As a Strategy to Find Early Predictive Signatures of Asthma in Children. *PLoS One* **2014**, *9*, e95668, doi:10.1371/journal.pone.0095668.

10. Phillips, M.; Gleeson, K.; Hughes, J.M.B.; Greenberg, J.; Cataneo, R.N.; Baker, L.; McVay, W.P. Volatile organic compounds in breath as markers of lung cancer: a cross-sectional study. *Lancet* **1999**, *353*, 1930–1933, doi:10.1016/S0140-6736(98)07552-7.

11. Phillips, M.; Cataneo, R.N.; Ditkoff, B.A.; Fisher, P.; Greenberg, J.; Gunawardena, R.; Kwon, C.S.; Tietje, O.; Wong, C. Prediction of breast cancer using volatile biomarkers in the breath. *Breast Cancer Res. Treat.* **2006**, *99*, 19–21, doi:10.1007/s10549-006-9176-1.

12. Gaspar, E.M.; Lucena, A.F.; Duro da Costa, J.; Chaves das Neves, H. Organic metabolites in exhaled human breath—A multivariate approach for identification of biomarkers in lung disorders. *J. Chromatogr. A* **2009**, *1216*, 2749–2756, doi:10.1016/j.chroma.2008.10.125.

13. Gaida, A.; Holz, O.; Nell, C.; Schuchardt, S.; Lavae-Mokhtari, B.; Kruse, L.; Boas, U.; Langejuergen, J.; Allers, M.; Zimmermann, S.; et al. A dual center study to

compare breath volatile organic compounds from smokers and non-smokers with and without COPD. *J. Breath Res.* **2016**, *10*, 026006, doi:10.1088/1752-7155/10/2/026006.

14. Ibrahim, B.; Basanta, M.; Cadden, P.; Singh, D.; Douce, D.; Woodcock, A.; Fowler, S.J. Non-invasive phenotyping using exhaled volatile organic compounds in asthma. *Thorax* **2011**, *66*, 804–809, doi:10.1136/thx.2010.156695.

15. Martinez-Lozano Sinues, P.; Meier, L.; Berchtold, C.; Ivanov, M.; Sievi, N.; Camen, G.; Kohler, M.; Zenobi, R. Breath Analysis in Real Time by Mass Spectrometry in Chronic Obstructive Pulmonary Disease. *Respiration* **2014**, *87*, 301–310, doi:10.1159/000357785.

16. Song, G.; Qin, T.; Liu, H.; Xu, G.-B.; Pan, Y.-Y.; Xiong, F.-X.; Gu, K.-S.; Sun, G.-P.; Chen, Z.-D. Quantitative breath analysis of volatile organic compounds of lung cancer patients. *Lung Cancer* **2010**, *67*, 227–231, doi:10.1016/j.lungcan.2009.03.029.

17. Dragonieri, S.; Schot, R.; Mertens, B.J.A.; Le Cessie, S.; Gauw, S.A.; Spanevello, A.; Resta, O.; Willard, N.P.; Vink, T.J.; Rabe, K.F.; et al. An electronic nose in the discrimination of patients with asthma and controls. *J. Allergy Clin. Immunol.* **2007**, *120*, 856–862, doi:10.1016/j.jaci.2007.05.043.

18. Oguma, T.; Nagaoka, T.; Kurahashi, M.; Kobayashi, N.; Yamamori, S.; Tsuji, C.; Takiguchi, H.; Niimi, K.; Tomomatsu, H.; Tomomatsu, K.; et al. Clinical contributions of exhaled volatile organic compounds in the diagnosis of lung cancer. *PLoS One* **2017**, *12*, e0174802, doi:10.1371/journal.pone.0174802.

19. Chen, X.; Xu, F.; Wang, Y.; Pan, Y.; Lu, D.; Wang, P.; Ying, K.; Chen, E.; Zhang, W. A study of the volatile organic compounds exhaled by lung cancer cells in vitro for breath diagnosis. *Cancer* **2007**, *110*, 835–844, doi:10.1002/cncr.22844.

20. Cazzola, M.; Segreti, A.; Capuano, R.; Bergamini, A.; Martinelli, E.; Calzetta, L.; Rogliani, P.; Ciaprini, C.; Ora, J.; Paolesse, R.; et al. Analysis of exhaled breath fingerprints and volatile organic compounds in COPD. *COPD Res. Pract.* **2015**, *1*, 7, doi:10.1186/s40749-015-0010-1.

21. van de Kant, K.D.G.; van Berkel, J.J.B.N.; Jobsis, Q.; Lima Passos, V.; Klaassen, E.M.M.; van der Sande, L.; van Schayck, O.C.P.; de Jongste, J.C.; van Schooten, F.J.; Derks, E.; et al. Exhaled breath profiling in diagnosing wheezy preschool children. *Eur. Respir. J.* **2013**, *41*, 183–188, doi:10.1183/09031936.00122411.

22. Van Berkel, J.J.B.N.; Dallinga, J.W.; Möller, G.M.; Godschalk, R.W.L.; Moonen, E.J.; Wouters, E.F.M.; Van Schooten, F.J. A profile of volatile organic compounds in breath discriminates COPD patients from controls. *Respir. Med.* **2010**, *104*, 557–563, doi:10.1016/j.rmed.2009.10.018.

23. Van Vliet, D.; Smolinska, A.; Jöbsis, Q.; Rosias, P.P.R.; Muris, J.W.M.; Dallinga, J.W.; van Schooten, F.J.; Dompeling, E. Association between exhaled inflammatory markers and asthma control in children. *J. Breath Res.* **2016**, *10*, 016014, doi:10.1088/1752-7155/10/1/016014.

24. Peng, G.; Hakim, M.; Broza, Y.Y.; Billan, S.; Abdah-Bortnyak, R.; Kuten, A.; Tisch, U.; Haick, H. Detection of lung, breast, colorectal, and prostate cancers from exhaled breath using a single array of nanosensors. *Br. J. Cancer* **2010**, *103*, 542–551, doi:10.1038/sj.bjc.6605810.
